# Activation of the sirtuin silent information regulator 1 pathway inhibits pathological myocardial remodeling

**DOI:** 10.3389/fphar.2023.1111320

**Published:** 2023-02-10

**Authors:** Youheng Wang, Rusheng Zhao, Chengyan Wu, Xuefei Liang, Lei He, Libo Wang, Xuehui Wang

**Affiliations:** ^1^ Department of Cardiology, The First Affiliated Hospital of Xinxiang Medical University, Heart Center of Xinxiang Medical University, Xinxiang, China; ^2^ Department of Cardiology, Guangyuan Central Hospital, Guangyuan, China; ^3^ College of Chemistry and Chemical Engineering, Henan Normal University, Xinxiang, China

**Keywords:** sirtuins, oxidative stress, myocardial remodeling, NLRP3 inflammasome, autophagy

## Abstract

Myocardial remodeling refers to structural and functional disorders of the heart caused by molecular biological changes in the cardiac myocytes in response to neurological and humoral factors. A variety of heart diseases, such as hypertension, coronary artery disease, arrhythmia, and valvular heart disease, can cause myocardial remodeling and eventually lead to heart failure. Therefore, counteracting myocardial remodeling is essential for the prevention and treatment of heart failure. Sirt1 is a nicotinamide adenine dinucleotide^+^-dependent deacetylase that plays a wide range of roles in transcriptional regulation, energy metabolism regulation, cell survival, DNA repair, inflammation, and circadian regulation. It positively or negatively regulates myocardial remodeling by participating in oxidative stress, apoptosis, autophagy, inflammation, and other processes. Taking into account the close relationship between myocardial remodeling and heart failure and the involvement of SIRT1 in the development of the former, the role of SIRT1 in the prevention of heart failure *via* inhibition of myocardial remodeling has received considerable attention. Recently, multiple studies have been conducted to provide a better understanding of how SIRT1 regulates these phenomena. This review presents the progress of research involving SIRT1 pathway involvement in the pathophysiological mechanisms of myocardial remodeling and heart failure.

## 1 Introduction

Cardiovascular diseases (CVDs) are the leading cause of death globally. According to the World Health Report, an estimated 17.9 million people died from CVD s in 2019, representing 32% of all global deaths. Out of the 17 million premature deaths (under the age of 70) due to non-communicable diseases in 2019, 38% were caused by CVDs. Heart failure is a type of CVD. Almost all types of heart failure are associated with myocardial remodeling. Myocardial remodeling is a compensatory process caused primarily by obesity, hypertension, heart valve disease, and cardiovascular disease. In the early phase, myocardial remodeling is characterized by the thickening of the ventricular wall and improvement in myocardial systolic function. However, in the long-term, myocardial remodeling is accompanied by interstitial fibrosis, systolic dysfunction, and abnormalities in gene expression, protein expression, energy metabolism, and electrophysiological characteristics, eventually leading to decompensated heart failure (Frey et al., 2004). The mechanisms of myocardial remodeling have not been fully elucidated and are mainly related to the activation of various cellular signaling pathways. The most well-known mechanisms of myocardial remodeling are related to the activation of the renin-angiotensin-aldosterone system, sympathetic stimulation, apoptosis, inflammation, oxidative stress, myocardial fibrosis, etc (Sciarretta et al., 2018). Sirtuin silent information regulator 1 (SIRT1) is a nicotinamide adenine dinucleotide (NAD^+^)-dependent histone deacetylase (HDAC) that plays an important role in biological processes, such as inflammation, apoptosis, and oxidative stress response (Hajializadeh and Khaksari, 2022). This article reviews the role and molecular mechanisms of Sirt1 protein in myocardial remodeling and outlines the specific mechanisms by which Sirt1 improves different types of myocardial remodeling and heart failure.

## 2 Sirtuins and cardiovascular regulation

Sirtuins are highly conserved class III histone deacetylases. Seven sirtuin-encoding genes (*SIRT1-7*) have been identified and characterized in mammals. They are localized in different cellular sites and play different roles: regulation of metabolism, oxidative stress, apoptosis, inflammation, and senescence. Sirtuins perform their functions by deacetylating target proteins at different sites. They play an important role in cardiovascular biology and may modulate cardiovascular health and age-dependent cardiovascular diseases (Cencioni et al., 2015), different members of the family playing different roles (Table 1). Recently, it has become clear that SIRT1 deacetylates histone and non-histone proteins to participate in multiple cellular process, including apoptosis, autophagy, calorie restriction, energy metabolism, transcriptional regulation, cell survival, DNA repair, inflammation, and circadian regulation (Luo et al., 2019). SIRT1 is not only an important regulatory mechanism involved in the pathologic occurrence of myocardial remodeling, but also involves many therapeutic targets for improving myocardial remodeling with drugs. Based on SIRT1, it has a broad prospect in revealing the pathological mechanism of myocardial remodeling and developing new clinical drugs.

**TABLE 1:** Type, location, and function of various sirtuins.

Sirtuins	Location	Model	Function
SIRT1	Cytoplasm, nucleus	Hypoxic mouse model	Promotes autophagy, inhibits apoptosis Luo et al. (2019)
		Ischemia/reperfusion injury	Inhibits apoptosis, reduces oxidative stress Zhang et al. (2018)
		Doxorubicin-induced cardiotoxicity	Reduces oxidative stress, inhibits apoptosis, improves the ejection function Hu et al. (2020)
			Inhibits inflammation and aging Zhang et al. (2021b)
		Myocardial hypertrophy	Reduces oxidative stress, inhibits apoptosis Ren et al. (2021)
SIRT2	Cytoplasm, nucleus	Doxorubicin-induced cardiotoxicity	Reduces oxidative stress Zhao et al. (2018a), Zhao et al. (2018b)
		Myocardial hypertrophy	Inhibits fibrosis, improves the ejection function Tang et al. (2017)
		Dilated cardiomyopathy	Inhibits inflammation Sun et al. (2022a)
SIRT3	Mitochondria	Sepsis-induced myocardial injury	Improves mitochondrial biogenesis Xin and Lu (2020)
		Myocardial hypertrophy	Reduces oxidative stress, improves endothelial dysfunction Dikalova et al. (2020)
SIRT4	Mitochondria	Ischemia/reperfusion injury	Inhibits apoptosis, improves mitochondrial biogenesis Zeng et al. (2018)
		Sirt 4^−/−^ rats	Reduces oxidative stress, promotes cardiac hypertrophy, promotes pulmonary fibrosis Luo et al. (2017)
		Doxorubicin-induced cardiotoxicity	Reduces apoptosis and autophagy He et al. (2022b)
SIRT5	Mitochondria	Heart failure	Inhibits inflammation, reduces oxidative stress, improves mitochondrial dysfunction Chang et al. (2021)
SIRT6	Nucleus	Hyperlipidemia	Inhibits inflammation, reduces atherosclerosis Grootaert et al. (2021)
		Diabetic cardiomyopathy	Increases autophagy, inhibits mitochondrial dysfunction Yu et al. (2021)
		Myocardial infarction	
		Doxorubicin-induced cardiotoxicity	Reduces oxidative stress, reduces apoptosis Wu et al. (2021)
		Aortic constriction-induced cardiopathy	Inhibits inflammation, reduces fibrosis, regulates telomere shortening Li et al. (2017)
SIRT7	Nucleus	Aortic constriction-induced cardiopathy	Reduces myocardial fibrosis, improves cardiac hypertrophy Yamamura et al. (2020)
		SIRT7^−/−^	Increases lifespan, inhibits inflammation Vakhrusheva et al. (2008)
			Promotes endothelial formation, increases smooth muscle proliferation Kimura et al. (2021)
		Hypoxia/reoxygenation injury *in vitro*	Reduces apoptosis Sun et al. (2018)

## 3 SIRT1-mediated apoptosis in pathological myocardial remodeling

Apoptosis is a distinct type of cell death characterized by a series of typical morphological events such as cell shrinkage, fragmentation into membrane-bound apoptotic vesicles, and rapid phagocytosis of neighboring cells without inducing an inflammatory response (Kerr et al., 1972). Apoptosis is thought to be the main cause of cell death within the first few hours after acute myocardial infarction (Palojoki et al., 2001). Sirt1 activates or inactivates apoptosis-associated proteins through deacetylation, thereby inhibiting apoptosis to ameliorate myocardial remodeling and delay the progression of heart failure. The activation of Sirt1 signaling pathway mediates apoptosis by various target signal.

### 3.1 Regulation of FoxO transcription factor

The transcription factor FoxO belongs to the forkhead protein O family, being one of the important transcription factors in the human body. It regulates the expression of genes that modulate glucose and lipid metabolism, oxidative stress, apoptosis, autophagy, and endoplasmic reticulum stress (Xing et al., 2018). It plays a key role in the development of cardiac remodeling (Zhang et al., 2021a). The effect of SIRT1 on FoxO function is complex and depends on FOXO target genes (Karbasforooshan and Karimi, 2017). Treatment with Ang II increased the acetylation of FoxO1 *in vivo* and *in vitro*, and subsequently the pro-apoptotic protein Bim was upregulated. Sirt1 overexpression deacetylated FoxO1, inhibiting Ang II-induced FoxO1 acetylation and reducing the level of the pro-apoptotic protein Bim. However, these beneficial effects were not observed after SIRT1 knockdown (Li et al., 2019b). Diabetic cardiomyopathy (DCM) is one of the main causes of myocardial remodeling. Notably, SIRT1-FoxO1 pathway was significantly inhibited in DCM mice, accompanied by increased myocardial apoptosis. So, Inhibition of SIRT1-FoxO1 pathway may be an important mechanism mediating diabetic induced cardiomyopathy. To prevent ambiguity, we have changed the description. Curcumin, a natural polyphenol isolated from turmeric root, has antioxidant, anti-inflammatory, anti-apoptotic, and anti-cancer effects. Curcumin partially restores SIRT1-FoxO1 pathway activity and inhibits apoptosis both *in vitro* and *in vivo.* Furthermore, the effect of curcumin on improving apoptosis disappeared after using EX527, a Sirt1 inhibitor (Ren et al., 2020). In addition, Sirt1 reduces p53 expression and apoptosis by targeting the FoxO3 transcription factor, and it deacetylates the DNA repair protein Ku70, which binds to and inactivates the pro-apoptotic factor Bax (Vahtola et al., 2008).

### 3.2 Regulation of the tumor suppressor gene p53

Overexpression of protein p53 owing to mutations of the tumor suppressor gene p53 leads to rapid loss of cell viability, which is characteristic of apoptosis. Increased levels of tumor suppressor p53 are associated with left ventricular hypertrophy and remodeling (Veeroju et al., 2020). MicroRNAs (miRNAs) are highly conserved, non-coding, single-stranded RNAs in eukaryotes. They can specifically recognize and complementarily bind to target mRNAs, leading to the post-transcriptional degradation of target genes (Henning, 2021). Downregulation of miR-128 in mice with heart failure significantly improved myocardial remodeling and counteracted Ang II-induced apoptosis, by targeting the SIRT1/p53 signaling pathway. Conversely, treatment with EX527 abolished the beneficial effect of miR-128 downregulation (Zhan et al., 2021). Taurine, a free intracellular β-amino acid, has cardioprotective effects. In mice with aortic constriction (TAC)-induced cardiomyocyte hypertrophy, compared to control, taurine significantly decreased the levels of acetylated (at Lys382) p53/p53. Taurine increased the NAD^+^/NADH ratio, promoted SIRT1 expression, inactivated p53 deacetylation, and inhibited cardiomyocyte apoptosis. Furthermore, EX527 mitigated the beneficial effects of taurine on cardiac function, levels of natriuretic peptide, and apoptosis (Liu et al., 2020). Anthracycline chemotherapeutic agents, such as doxorubicin (DOX), cause cardiotoxicity. DOX treatment leads to apoptosis through p53 activation. Thus, by regulating the levels of p53 expression, SIRT1 activation significantly reduces DOX-induced fibrosis, hypertrophy, and apoptosis in H9c2 cardiomyocytes (Lohanathan et al., 2022). CTRP3 preserves DOX-induced cardiac dysfunction and alleviates DOX-induced cardiac inflammation and apoptosis by activating SIRT1, So, sirt1 is an important therapeutic target for correcting DOX induced cardiac remodeling (Yuan et al., 2018).

### 3.3 Upregulation of SIRT1 expression *via* adenosine 5′-monophosphate-activated protein kinase (AMPK) modulates apoptosis

AMPK is considered cellular fuel. Cellular energy is a key regulator of the AMPK system (Carling, 2017). The cellular energy imbalance that occurs during stress leads to an increase in the intracellular AMP/ATP ratio, resulting in AMPK activation. This helps cells survive without oxygen (Horman et al., 2012). In myocardial tissue, under pathological conditions, such as hyperglycemia, hypoxia, and pressure overload, AMPK activation is significantly downregulated (Li et al., 2019a; Zhang et al., 2020). In diabetic rats, treatment with metformin and atorvastatin reduced the levels of expression of caspase-3 and increased the Bcl-2/Bax ratio. Furthermore, in diabetic rats, the levels of expression of p-AMPK and Sirt1 were downregulated, and this was reversed by the combination treatment, which subsequently increased NAD^+^ levels and reduced apoptosis in cardiomyocytes (Jia et al., 2021; Laddha and Kulkarni, 2021). It's worth noting that AMPK is a trimeric serine/threonine protein kinase comprising a catalytic α subunit and non-catalytic *β* and *γ* subunits, which are encoded by seven different high-homologous genes (α1, α2, β1, β2, γ1, γ2, γ3). However, in the existing literature, the differences in the regulation of SIRT1 by AMPA coding from different high-homologous genes have not been clarified.

## 4 SIRT1 regulates oxidative stress in pathological myocardial remodeling

An imbalance between the production of ROS and the ability of the body to detoxify reactive intermediates results in oxidative stress. Prolonged pathological stimulation, release of inflammatory cytokines, and activation of mitogen-activated protein kinases lead to the generation of high levels of oxygen free radicals. Detoxification of ROS is ensured by the activity of antioxidant enzymes such as Mn-SOD, catalase, glutathione reductase, and peroxidase (Rababa’h et al., 2018). Oxidative stress is a major stimulus for signal transduction in cardiac myocytes. It causes abnormal Ca^2+^ metabolism associated with subcellular remodeling, defective energy production, inflammation, apoptosis, fibrosis, and cardiomyocyte loss, all of which are thought to contribute to myocardial remodeling and heart failure (Shah et al., 2021). Sirt1 suppresses oxidative stress, preventing pathological myocardial remodeling by.

### 4.1 Regulation of FoxO1 transcription factors

In an angiotensin-induced hypertension model, SIRT1 overexpression attenuated Ang II-induced ROS formation. Sirt1-mediated deacetylation of FoxO1 directly controlled the expression of catalase and MnSOD, thereby promoting the breakdown of ROS and suppressing oxidative stress (Li et al., 2019b). In H9C2 cells, Ang II increased the concentration of malondialdehyde and decreased SOD activity, thus inducing oxidative stress. Furthermore, it reduced the levels of expression of SIRT1 and FoxO1. Conversely, addition of a SIRT1 agonist attenuated the Ang II-induced oxidative stress index (Jiang et al., 2021). Fibroblast growth factor 20 (FGF20) is a member of the fibroblast growth factor family and is involved in apoptosis, senescence, inflammation, and autophagy. FGF20 upregulates SIRT1 expression, leading to FOXO1 deacetylation, which promotes the transcription of downstream antioxidant genes, thereby suppressing oxidative stress. It has anti-hypertrophic effect which is greatly counteracted in SIRT1 knockout mice. Furthermore, these mice also presented an increase in oxidative stress (Chen et al., 2022b).

### 4.2 Regulation of NF-κB

NF-κB, a classical signaling pathway, is involved in the development of myocardial remodeling, and inhibition of its phosphorylation can help treat myocardial hypertrophy (Yu et al., 2013). A major component of ginseng, ginsenoside Rg3, has anti-aging effects [27] and ameliorates Ang-II-induced myocardial remodeling. The underlying mechanism consists of the regulation of the Sirt1/NF-κB pathway leading to downregulation of the expression of superoxide dismutase, malondialdehyde, heme oxygenase-1 (HO-1), and nuclear factor (erythroid-derived 2)-like2 (Nrf2). This reduction of oxidative stress ameliorates myocardial remodeling, and the phenomenon is counteracted by the administration of Sirt1 inhibitor AGK2 (Ren et al., 2021).

### 4.3 Regulation of transforming growth factor beta (TGF-β)

In an animal model of DCM, Sirt1 decreased the activity of TGF-β and prevented myocardial remodeling by inhibiting p300. Sirt1 deacetylates p65 subunit of NF-kB, leading to reduced binding of NF-KB-P65 to DNA. Subsequently, this reduces cardiac hypertrophy and oxidative stress by diminishing the transcription of subunits of NADPH oxidase (NOX1 and NOX2) (Karbasforooshan and Karimi, 2017). Epithelial mesenchymal transition (EndMT) is closely associated with pathogenesis of myocardial remodeling. EndMT is strongly induced by TGF-β. SIRT1 pathway inhibits EndMT by inhibiting the TGFβ/Smad pathway, thereby reducing cardiac fibrosis and reversing myocardial remodeling (Liu et al., 2019). Thus, ellagic acid, a phytochemical found mainly in nuts and some fruits (e.g., raspberries, grapes, and pomegranates), activated SIRT1 and inhibited TGF-β, suppressing oxidative stress and inhibiting myocardial remodeling caused by DCM (Altamimi et al., 2020). TGF-β promotes the development of fibrotic disease by enhancing collagen expression and inducing cell differentiation into myofibroblasts. And SIRT1 binding to Smad2/3 inhibited Smad2/3 nuclear translocation, thereby regulating myocardial remodeling *via* the TGF-β/Smad2/3 pathway in cardiac fibroblasts (Liu et al., 2019).

### 4.4 Regulation of the expression of peroxisome proliferator-activated receptor gamma coactivator 1 (PGC-1)

Mitochondrial biogenesis is a central player in the pathophysiology of many cardiovascular diseases, including myocardial remodeling (Forte et al., 2021). Oxidative stress is closely associated with mitochondrial biogenesis. One major regulator of this phenomenon is PGC-1α which is activated by Sirt1 through deacetylation. Subsequently, mitochondrial function improves and ROS production is reduced (Waldman et al., 2018). Neuraminidase-1 (NEU1) is involved in the response to multiple signals and regulates a variety of cellular metabolic processes. Furthermore, it is closely associated with the onset and progression of cardiovascular disease. NEU1 knockdown attenuates cardiomyocyte injury by regulating the SIRT1/PGC-1α signaling pathway, thereby promoting mitochondrial biogenesis and function. Canagliflozin is used to treat type 2 diabetes and improves myocardial remodeling, and therefore being recommended in heart failure guidelines. This effect is mediated by the activation of the AMPK/SIRT1/PGC-1α signaling pathway, upregulating PGC-1α expression and reducing cardiac hypertrophy, fibrosis, and oxidative stress (He et al., 2022a).

Additionally, the levels of expression of SIRT1 are modulated by AMPK, which thus, is implicated in the control of ROS levels. During the development of cardiac remodeling, energy deficits can exacerbate cardiac insufficiency. Elevated expression of antioxidants (SOD1, catalase, and MnSOD) and reduced mitochondrial ROS production mitigates myocardial remodeling. AMPK is an upstream regulator of Sirt1. As mentioned before, in reality, AMPK and Sirt1 regulate each other and share many common target molecules; AMPK increases NAD^+^ levels and activates Sirt1 (Wang et al., 2020b). The expression levels of p-AMPK and SIRT1 were reduced in diabetic mice and in H9C2 cells exposed to high concentrations of glucose. Combined treatment with metformin and atorvastatin activated the AMPK/SIRT1 signaling pathway, thereby attenuating cardiomyocyte fibrosis, hypertrophy, and oxidative stress (Jia et al., 2021). Aldehyde dehydrogenase 2 (ALDH2) is an essential mitochondrial enzyme that controls cardiac function. It exacerbates aging-induced cardiac hypertrophy, oxidative stress, and mitochondrial damage. AMPK/SIRT1 activation (resveratrol and SRT1720) prevented ALDH2-induced contractile dysfunction in cardiomyocytes. AMPK enhances SIRT1 activity by increasing cellular NAD^+^ levels, leading to deacetylation and regulation of the activity of downstream targets of SIRT1, including PGC1α, thereby reducing oxidative stress in cardiomyocytes (Zhang et al., 2014).

In summary, the main molecular mechanism of SIRT1-mediated regulation of oxidative stress is the modulation of the levels of expression of catalase and MnSOD through SIRT1-mediated FoxO1 deacetylation, which leads to the inhibition of the production of ROS and suppression of oxidative stress. PGC-1α and NF-κB are activated by SIRT1 through deacetylation, thereby improving mitochondrial function and counteracting the pro-oxidant effect of cell stress, thereby reversing cardiomyocyte remodeling.

## 5 SIRT1 signaling mediates inflammatory responses in pathological myocardial remodeling

Inflammatory cytokines play an important role in the pathophysiology of adverse myocardial remodeling and are significantly elevated in both heart failure and adverse myocardial remodeling (Hanna and Frangogiannis, 2020). The early inflammatory response after myocardial infarction may increase myocardial fibrosis and remodeling (Gao et al., 2019). The prevalence of DCM in diabetes is approximately 17%, and patients with type 1 and type 2 diabetes have a significantly increased risk of heart failure. DCM progresses in part through inflammation, leading to structural changes in the diabetic heart (Elmadbouh and Singla, 2021). SIRT1 signaling mediates inflammatory responses in pathological myocardial remodeling by various mechanisms, the main one being regulation of transcriptional co-activator PGC-1a. Obese mice show significant cardiac hypertrophy, inflammatory cell infiltration, reduced SIRT1 activity, altered mitochondrial signaling and oxidative homeostasis, and overexpression of inflammatory markers. Melatonin prevents cardiac remodeling caused by obesity by activating SIRT1, which regulates cellular metabolic signaling by acetylating and activating the coactivator PGC-1α. This induces mitochondrial transcription factors, thereby enhancing mitochondrial content and cellular metabolic oxidative capacity, reducing oxidative stress and inflammation. Nrf2, a transcription factor regulated by PGC-1α, is significantly reduced in the hearts of obese mice, leading to a significant decrease in the levels of HO-1 expression and an increase in lipid peroxidation and the levels of the pro-inflammatory markers NLRP3, tumor necrosis factor-α, and IL-6. The SIRT1/PGC-1α/Nrf2/HO-1 pathway is a key for preventing adverse obesogenic myocardial remodeling (Favero et al., 2020). Tongguan capsule dramatically decreased the expressions of TNF-α, IL-1β, and IL-6 Post-myocardial Infarction Remodeling through Sirt1 Activation. And the induction of Sirt1 by TGC was inhibited by the specific inhibitor EX527. In the presence of EX527, TGC-induced autophagy-specific proteins were down-regulated, while inflammatory factors were upregulated (Mao et al., 2018). And in streptothromycin-induced diabetic mouse models, phloetin exerts anti-inflammatory effects by docking with SIRT1, thereby protecting against cardiac injury and remodeling (Ying et al., 2019). Therefore, SIRT1-mediated inflammatory response is also an important therapeutic target for myocardial remodeling.

## 6 SIRT1 signaling regulates cellular autophagy in pathological myocardial remodeling

Besides necrosis and apoptosis, autophagy is another type of cellular death (Glick et al., 2010). Autophagy involves the formation of autophagic vesicles, which encapsulate degraded or long-lived proteins and organelles and then fuse with lysosomes. Autophagy plays multiple roles in myocardial hypertrophy. In hypertrophic cardiomyocytes, autophagy can ensure the degradation of excess harmful substances, reduce cytotoxic damage caused by misfolded protein aggregation, mitigate oxidative stress, and maintain cell survival. Because cardiomyocytes are terminally differentiated cells, their survival is overly dependent on autophagy to self-clean abnormal substances; therefore, effective autophagy is essential for the stability of the cardiovascular internal environment (Bravo-San Pedro et al., 2017). Increasing evidence suggests that dysregulation of cardiomyocyte autophagy is associated with the progression of myocardial remodeling (Shirakabe et al., 2016). Excessive autophagy can exacerbate mitochondrial damage and impair energy metabolism by non-selectively degrading normal mitochondria and mitochondria-associated proteins, thereby resulting in energy disorders. In summary, basal levels of autophagy are essential for ensuring a proper functioning of cardiomyocytes and dysregulated autophagy can lead to cardiomyocyte hypertrophy (Zheng et al., 2021). SIRT1 signaling regulates cellular autophagy in pathological myocardial remodeling by various mechanisms.

### 6.1 Interaction with AMPK

Among drugs that prevent myocardial remodeling, angiotensin-converting enzyme inhibitors (ACEIs) are commonly used in clinical practice. Furthermore, they promote autophagy in cardiomyocytes (Wu et al., 2013) owing to the activation of the AMPK pathway (Hernández et al., 2014). Metformin is an activator of AMPK and is used clinically for the treatment of diabetes. It can reduce the serious complications of diabetes, including myocardial remodeling and heart failure by upregulating SIRT1 and AMPK and subsequently, promoting autophagy (Xie et al., 2011). Sodium-glucose cotransporter (SGLT2) is not expressed in the heart but SGLT2 inhibitors are recommended by the latest treatment guidelines in patients with heart failure, with or without diabetes mellitus. SGLT2 inhibitors may directly bind to SIRT1, thus inhibiting autophagy (Osataphan et al., 2019).

### 6.2 Regulation of FoxO1 transcription factor

Treatment of rat cardiomyocyte cell lines with Ang II results in insufficient cardiomyocyte autophagy and interferes with the expression of the autophagy-associated proteins beclin1 and p62. Ginkgolide B Protects Cardiomyocytes from Angiotensin II-Induced Hypertrophy *via* Regulation of Autophagy through SIRT1-FoxO1 (Jiang et al., 2021). Another study showed that SIRT1-dependent deacetylation of the transcription factor Foxo1 is involved in cardiac senescence: SIRT1 activates FoxO1, promoting its nuclear localization, and Akt inhibits it by phosphorylation, preventing nuclear translocation. The inhibition of SIRT1-Foxo1-mediated autophagy in aged mice can be counteracted by the ablation of Akt2, an enzyme that has the opposite effect (Ren et al., 2017).

### 6.3 Modulation of FGF21 expression

FGF21 is a novel peptide ligand involved in a variety of physiological and pathological processes, including regulation of glucose and lipid metabolism and reduction of atherosclerotic plaque formation in large blood vessels. It also plays a cardioprotective role in myocardial infarction, cardiac ischemia-reperfusion injury, cardiac hypertrophy, and DCM (Geng et al., 2020). In animal models of DCM, diabetes-induced oxidative damage and inflammation inhibited cardiac autophagy, suggesting that these phenomena contribute to the pathogenesis of diabetic heart disease (Zhang et al., 2016). Fenofibrate (FF), a peroxisome proliferator-activated receptor-α (PPARα) agonist, is used clinically to treat hypertriglyceridemia. In a mouse model of diabetic cardiomyopathy, pre-treatment with FF partially restored autophagy in diabetic hearts. Furthermore, the cardioprotective effect of FF in type 1 diabetes mellitus is dependent on FGF21. The upregulation of cardiac FGF21 expression may increase SIRT1-mediated autophagy, which plays a key role in preventing diabetes-induced cardiac inflammation, oxidative stress, fibrosis, and dysfunction (Zhang et al., 2016). *In vitro* studies using H9C2 cells also showed that exposure to high glucose (HG) significantly increased inflammatory responses, oxidative stress, and pro-fibrotic responses, and significantly inhibited autophagy. The effects of HG were inhibited by treatment with FF. Inhibition of autophagy by 3-methyladenine (3 MA) or SIRT1 by sirtinol (SI) counteracted the beneficial effects of FF. Thus, FF may prevent cardiac pathology and functional abnormalities caused by type 1 diabetes by increasing FGF21 levels, which may upregulate SIRT1-mediated autophagy. Moreover, FGF21 is involved in multiple disease processes through SIRT1-dependent autophagy, including wound healing (Chen et al., 2022a), osteoarthritis (Lu et al., 2021b), and acute liver injury (Yang et al., 2022), which overlaps with the pathological mechanism of myocardial remodeling So, SIRT1-dependent autophagy mediated by FGF21 may be an important mechanism involved in pathological myocardial remodeling.

In summary, SIRT1-dependent deacetylation of FoxO1 prevents cardiac senescence and enhances cellular autophagy. SIRT1 plays a key role in preventing diabetes-induced cardiac inflammation, oxidative stress, and dysfunction. Ang-II induces cardiac hypertrophy by suppressing SIRT1 expression, whereas ginkgolide B counteracts it by enhancing autophagy through the activation of the SIRT1-FoxO1 pathway.

## 7 SIRT1 improves mitochondrial dysfunction mitigating myocardial remodeling

The heart is the most metabolically active organ, accounting for approximately 8% of ATP consumption daily (Brown et al., 2017). Impaired mitochondrial function is involved in the development and progression of maladaptive cardiac hypertrophy and heart failure (Tham et al., 2015). In myocardial remodeling and heart failure, mitochondrial production of energy gradually decreases. This leads to increased production of ROS and cytoplasmic release of cytochrome c, which promote programmed cell death, cardiomyocyte injury, and ultimately, heart failure (Elorza and Soffia, 2021). Histidine attenuates pressure overload and phenylephrine (PE)-induced myocardial hypertrophy through upregulation of SIRT1, which prevents mitochondrial dysfunction and oxidative damage in response to hypertrophic stimuli and maintains mitochondrial respiratory function and ATP synthesis. Inhibition of SIRT1 could reverse the protective effect of histidine on myocardial hypertrophy (Wang et al., 2020b). SIRT1 was shown to regulate mitochondrial energy transduction, ATP synthesis, and biogenesis by upregulating the activity of PPARα and PGC-1 (Planavila et al., 2011).

ATP deficiency can cause myocardial contractile dysfunction, whereas adenosine monophosphate activated protein kinase (AMPK) activity is regulated by the ADP/ATP ratio. SIRT1 is required for the activity of AMPK (Price et al., 2012). It has been demonstrated that activation of SIRT1 can prevent the decrease in ATP while promoting the transcription of energy metabolism-related genes (Um et al., 2010). Meahwile, Sirt1 stimulates the ability of PGC-1α to coactivate hepatocyte nuclear factor 4αand to inhibit glycolytic genes in response to pyruvate, thereby positively regulating gluconeogenic genes in response to pyruvate in hepatic cells (Rodgers et al., 2005). Additionally, Loss of Sirt1 activity led to dilated cardiomyopathy in adult hearts, which is accompanied by mitochondrial dysfunction (Planavila et al., 2012). However, another study has shown that Sirt1 is upregulated in failing hearts and inhibits the expression of genes associated with mitochondrial function (Oka et al., 2011). Therefore, the effects of Sirt1 on cardiac mitochondrial function and metabolism are also complex and may be dose-dependent or even bidirectional.

## 8 Inducers and inhibitors of SIRT1

### 8.1 Inducers of SIRT1

Since SIRT1 and its regulation play an important role in human diseases, there is an increasing interest in discovering small molecules that modulate its activity. Common agonists of SIRT1 are reviewed here. The polyphenol resveratrol (RSV), a natural compound, was the first SIRT1 agonist. Resveratrol exhibits a wide range of physiological and biochemical activities, including antioxidant, anti-inflammatory, antiplatelet, and anticoagulant activities, suggesting that its administration is beneficial for cardiovascular diseases (Bonnefont-Rousselot, 2016). RSV inhibits cell membrane lipid oxidation, protects low-density lipoprotein from oxidation, and increases the concentration of high-density lipoprotein (Berrougui et al., 2009). RSV has antithrombotic effects and inhibits thrombosis by inhibiting prostaglandin and thromboxane synthesis and platelet activity (Snopek et al., 2018). In the cardiovascular system, disturbances in intracellular calcium homeostasis lead to cardiovascular system dysfunction, including cardiac systolic dysfunction, arrhythmias, remodeling, and apoptosis. RSV differentially regulates Ca^2+^ handling by stimulation of NO production or antioxidant activity, maintaining Ca^2+^ homeostasis under normal and pathological conditions (Liu et al., 2017). Other SIRT1 agonists include small molecules that are structurally different from RSV, but hundreds of times more potent, such as SRT1720 and SRT501.

### 8.2 SIRT1 inhibitors

SIRT1 agonists are considered beneficial in a variety of diseases, as demonstrated in various animal models. However, SIRT1 inhibitors might be beneficial in cancer, Parkinson’s disease, and infection with human immunodeficiency virus (HIV) (Pagans et al., 2005; Alcaín and Villalba, 2009). Sirtinol induces senescence-like growth arrest in human breast cancer MCF-7 and lung cancer H1299 cells (Ota et al., 2006). Sirt1 activation promotes chronic granulocytic leukemia (CML) cell survival, and proliferation is associated with the deacetylation of multiple SIRT1 substrates, including FoxO1, p53, and KU70. Treatment of mice with the SIRT1 inhibitor tenovin-6 prevents cancer progression (Yuan et al., 2012). Table 2 shows both the inducers and inhibitors of SIRT1.

**TABLE 2:** Inducers and inhibitors of Sirt-1.

Number	Name	Data source	References
Sirt1 inducers			
1	Resveratrol	A review	Bhullar and Hubbard (2015)
2	Salvianolic acid	A rat model of chronic alcoholic liver disease	Zhang et al. (2017b)
3	Quercetin	A rat model of osteoarthritis	Qiu et al. (2018)
4	Fisetin	3T3-L1 cells model	Kim et al. (2015)
5	Panaxtriol saponins	PC12 cells and zebrafish	Zhang et al. (2017a)
6	Ginsenoside Rg3	Aged rats model	Yang et al. (2018)
7	Ginsenoside Rb2	H9C2 cell line	Huang et al. (2019)
8	Ginsenoside Rc	HEK293T cell line	Kim et al. (2014)
9	Ophiopogonin D	H9c2 cell line	Wang et al. (2016)
10	SRT1720	Obese mice model	Minor et al. (2011)
11	Strigolactone analogue GR24	Rat L6 skeletal muscle cell line	Modi et al. (2017)
12	SIRT1460	Obese mice model	Milne et al. (2007)
13	SRT2183	sirt1^−/−^ mice model	Gurt et al. (2015)
14	A03	Alzheimer’s disease mouse model	Campagna et al. (2018)
15	MHY2233	db/db mice model	Kim et al. (2018)
16	SRT2104	Clinic trial	Lin et al. (2019)
17	SRT3025	Clinic trial	Venkatasubramanian et al. (2013)
Sirt1 inducers			
1	Sirtinol	Rats model	Safari et al. (2017)
2	cambinol	RPMI8226 and U266 cells	Lu et al. (2021a)
3	inauhzin	HCT116 and DLD1 cells	Sun et al. (2022b)
4	EX527	U87MG and LN-299 glioma cell lines	Wang et al. (2020a)
5	AGK2	A549 and H1299 non-small cell lung cancer cells	Ma et al. (2018)
6	Suramin	Structure–activity Study	Trapp et al. (2007)
7	Tenovin	HCT116 cells	Ueno et al. (2013)
8	Salermide	BxPC-3 pancreatic cancer cell line	Yar Saglam et al. (2016)

SIRT1 activators have been proposed as a therapeutic strategy for treating and preventing vascular disease. A clinical trial proved that the defective Sirt1 may be correlated to the abnormal IFNγ expression in severe aplastic anemia patients, and activation of Sirt1 signaling by SRT3025 may help improve the inflammatory status of severe aplastic anemia (Lin et al., 2019). And Sowmya found SRT2104, an activator of SIRT1, appears to be safe and well tolerated and associated with an improved lipid profile without demonstrable differences in vascular or platelet function in otherwise healthy cigarette smokers based on a double-blind trial (Venkatasubramanian et al., 2013). At present, more ongoing clinical trials areunderway to investigate the efficacy, pharmacokinetics, and safety of Sirtuin modulator compounds inseveral diseases (http://clinicaltrials.gov). Unfortunately, there are still reports about the use of sirt modulators in clinical trials. So, future research should aim to elucidate the role of Sirt1 completely and to develop pharmacological strategies that can allow its action to be modulated (Table 2).

## 9 Discussion

**FIGURE 1:**
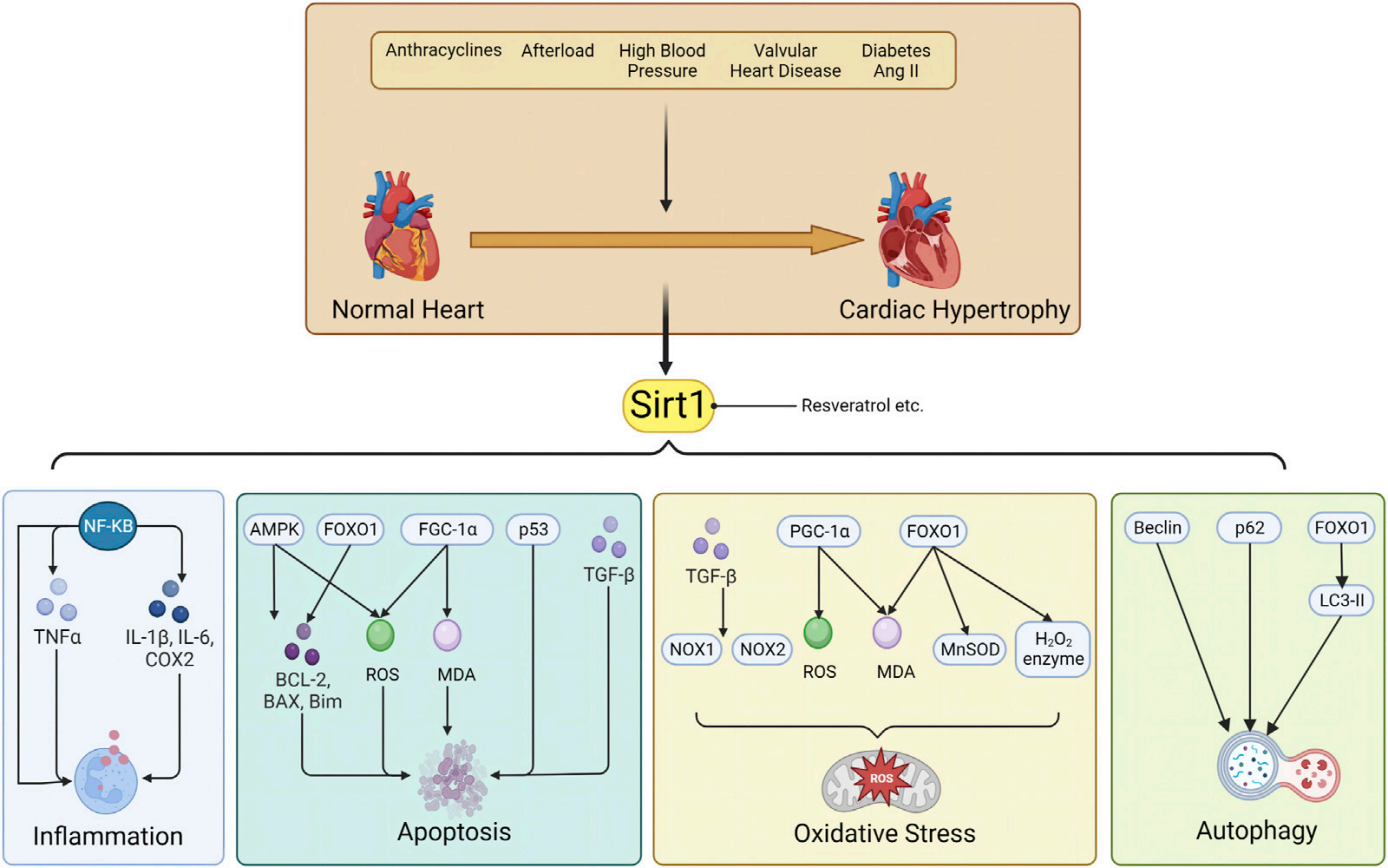
Sirt1 is involved in the pathological mechanism of myocardial remodeling through inflammation, apoptosis, oxidative stress and autophagy.

(4) Myocardial remodeling is a common pathophysiological process in heart failure, and its amelioration is a cornerstone of chronic heart failure treatment. However, drugs capable of reversing myocardial remodeling are scarce; thus, representing a current clinical unmet need. SIRT1 plays a key role in the pathogenesis of cardiovascular diseases. It mediates oxidative stress, apoptosis, autophagy, inflammation, and mitochondrial dysfunction in cardiomyocytes. Its activation reverses myocardial remodeling and other cardiac diseases, such as coronary atherosclerosis, which accelerates the onset of heart failure and increases heart failure-related morbidity and mortality. We have summarized the role of SIRT1 in cardiac remodeling of various etiologies, and the underlying mechanisms, including 1) SIRT1-mediated apoptosis by FoxO transcription factor, p53 and AMPK pathway; 2) SIRT1 regulates oxidative stress by FoxO1 transcription factors, NF-κB, TGF-β and PGC-1 pathway. 3) SIRT1 regulates inflammatory responses through acetylation and activation of the coactivator PGC-1α; 4) SIRT1 signaling regulates cellular autophagy by interaction with AMPK, regulation FoxO1 transcription factor and modulation of FGF21 activity. The specific molecular pathways involved are shown in Figure 1.

Although the specific mechanisms of myocardial remodeling have not yet been fully elucidated, some critical elements have been identified. These might represent relevant therapeutic targets that are associated with the pathogenesis of myocardial remodeling. Preclinical data indicate that SIRT1 is a promising target - increasing evidence suggests that SIRT1 activation ameliorates or prevents myocardial remodeling, delaying the progression of heart failure.

## References

[B1] Alcaín F. J. Villalba J. M. (2009). Sirtuin inhibitors. Expert Opin. Ther. Pat. 19, 283–294. 10.1517/13543770902755111 19441904

[B2] Altamimi J. Z. Alfaris N. A. Alshammari G. M. Alagal R. I. Aljabryn D. H. Aldera H. (2020). Ellagic acid protects against diabetic cardiomyopathy in rats by stimulating cardiac silent information regulator 1 signaling. J. physiology Pharmacol. 71. 10.26402/jpp.2020.6.12 33901999

[B3] Berrougui H. Grenier G. Loued S. Drouin G. Khalil A. (2009). A new insight into resveratrol as an atheroprotective compound: Inhibition of lipid peroxidation and enhancement of cholesterol efflux. Atherosclerosis 207, 420–427. 10.1016/j.atherosclerosis.2009.05.017 19552907

[B4] Bhullar K. S. Hubbard B. P. (2015). Lifespan and healthspan extension by resveratrol. Biochimica biophysica acta 1852, 1209–1218. 10.1016/j.bbadis.2015.01.012 25640851

[B5] Bonnefont-Rousselot D. (2016). Resveratrol and cardiovascular diseases. Nutrients 8, 250. 10.3390/nu8050250 27144581 PMC4882663

[B6] Bravo-San Pedro J. M. Kroemer G. Galluzzi L. (2017). Autophagy and mitophagy in cardiovascular disease. Circulation Res. 120, 1812–1824. 10.1161/circresaha.117.311082 28546358

[B7] Brown D. A. Perry J. B. Allen M. E. Sabbah H. N. Stauffer B. L. Shaikh S. R. (2017). Expert consensus document: Mitochondrial function as a therapeutic target in heart failure. Nat. Rev. Cardiol. 14, 238–250. 10.1038/nrcardio.2016.203 28004807 PMC5350035

[B8] Campagna J. Spilman P. Jagodzinska B. Bai D. Hatami A. Zhu C. (2018). A small molecule ApoE4-targeted therapeutic candidate that normalizes sirtuin 1 levels and improves cognition in an Alzheimer's disease mouse model. Sci. Rep. 8, 17574. 10.1038/s41598-018-35687-8 30514854 PMC6279743

[B9] Carling D. (2017). AMPK signalling in health and disease. Curr. Opin. cell Biol. 45, 31–37. 10.1016/j.ceb.2017.01.005 28232179

[B10] Cencioni C. Spallotta F. Mai A. Martelli F. Farsetti A. Zeiher A. M. (2015). Sirtuin function in aging heart and vessels. J. Mol. Cell. Cardiol. 83, 55–61. 10.1016/j.yjmcc.2014.12.023 25579854

[B11] Chang X. Zhang T. Wang J. Liu Y. Yan P. Meng Q. (2021). SIRT5-Related desuccinylation modification contributes to quercetin-induced protection against heart failure and high-glucose-prompted cardiomyocytes injured through regulation of mitochondrial quality surveillance. Oxidative Med. Cell. Longev. 2021, 5876841. 10.1155/2021/5876841 PMC8486530 34603599

[B12] Chen X. Tong G. Fan J. Shen Y. Wang N. Gong W. (2022a). FGF21 promotes migration and differentiation of epidermal cells during wound healing via SIRT1-dependent autophagy. Br. J. Pharmacol. 179, 1102–1121. 10.1111/bph.15701 34608629

[B13] Chen Y. An N. Zhou X. Mei L. Sui Y. Chen G. (2022b). Fibroblast growth factor 20 attenuates pathological cardiac hypertrophy by activating the SIRT1 signaling pathway. Cell death Dis. 13, 276. 10.1038/s41419-022-04724-w 35351862 PMC8964679

[B14] Dikalova A. E. Pandey A. Xiao L. Arslanbaeva L. Sidorova T. Lopez M. G. (2020). Mitochondrial deacetylase Sirt3 reduces vascular dysfunction and hypertension while Sirt3 depletion in essential hypertension is linked to vascular inflammation and oxidative stress. Circulation Res. 126, 439–452. 10.1161/circresaha.119.315767 31852393 PMC7035170

[B15] Elmadbouh I. Singla D. K. (2021). BMP-7 attenuates inflammation-induced pyroptosis and improves cardiac repair in diabetic cardiomyopathy. Cells 10, 2640. 10.3390/cells10102640 34685620 PMC8533936

[B16] Elorza A. A. Soffia J. P. (2021). mtDNA heteroplasmy at the core of aging-associated heart failure. An integrative view of OXPHOS and mitochondrial life cycle in cardiac mitochondrial physiology. Front. cell Dev. Biol. 9, 625020. 10.3389/fcell.2021.625020 33692999 PMC7937615

[B17] Favero G. Franco C. Stacchiotti A. Rodella L. F. Rezzani R. (2020). Sirtuin1 role in the melatonin protective effects against obesity-related heart injury. Front. physiology 11, 103. 10.3389/fphys.2020.00103 PMC7078333 32218740

[B18] Forte M. Schirone L. Ameri P. Basso C. Catalucci D. Modica J. (2021). The role of mitochondrial dynamics in cardiovascular diseases. Br. J. Pharmacol. 178, 2060–2076. 10.1111/bph.15068 32294237

[B19] Frey N. Katus H. A. Olson E. N. Hill J. A. (2004). Hypertrophy of the heart: A new therapeutic target? Circulation 109, 1580–1589. 10.1161/01.Cir.0000120390.68287.Bb 15066961

[B20] Gao R. Shi H. Chang S. Gao Y. Li X. Lv C. (2019). The selective NLRP3-inflammasome inhibitor MCC950 reduces myocardial fibrosis and improves cardiac remodeling in a mouse model of myocardial infarction. Int. Immunopharmacol. 74, 105575. 10.1016/j.intimp.2019.04.022 31299609

[B21] Geng L. Lam K. S. L. Xu A. (2020). The therapeutic potential of FGF21 in metabolic diseases: From bench to clinic. Nat. Rev. Endocrinol. 16, 654–667. 10.1038/s41574-020-0386-0 32764725

[B22] Glick D. Barth S. Macleod K. F. (2010). Autophagy: Cellular and molecular mechanisms. J. pathology 221, 3–12. 10.1002/path.2697 PMC2990190 20225336

[B23] Grootaert M. O. J. Finigan A. Figg N. L. Uryga A. K. Bennett M. R. (2021). SIRT6 protects Smooth muscle cells from senescence and reduces atherosclerosis. Circulation Res. 128, 474–491. 10.1161/circresaha.120.318353 33353368 PMC7899748

[B24] Gurt I. Artsi H. Cohen-Kfir E. Hamdani G. Ben-Shalom G. Feinstein B. (2015). The Sirt1 activators SRT2183 and SRT3025 inhibit RANKL-induced osteoclastogenesis in bone marrow-derived macrophages and down-regulate Sirt3 in Sirt1 null cells. PLoS One 10, e0134391. 10.1371/journal.pone.0134391 26226624 PMC4520518

[B25] Hajializadeh Z. Khaksari M. (2022). The protective effects of 17-β estradiol and SIRT1 against cardiac hypertrophy: A review. Heart Fail. Rev. 27, 725–738. 10.1007/s10741-021-10171-0 34537933

[B26] Hanna A. Frangogiannis N. G. (2020). Inflammatory cytokines and chemokines as therapeutic targets in heart failure. Cardiovasc. drugs Ther. 34, 849–863. 10.1007/s10557-020-07071-0 32902739 PMC7479403

[B27] He L. Ma S. Zuo Q. Zhang G. Wang Z. Zhang T. (2022a). An effective sodium-dependent glucose transporter 2 inhibition, canagliflozin, prevents development of hypertensive heart failure in dahl salt-sensitive rats. Front. Pharmacol. 13, 856386. 10.3389/fphar.2022.856386 35370704 PMC8964360

[B28] He L. Wang J. Yang Y. Zou P. Xia Z. Li J. (2022b). SIRT4 suppresses doxorubicin-induced cardiotoxicity by regulating the AKT/mTOR/Autophagy pathway. Toxicology 469, 153119. 10.1016/j.tox.2022.153119 35134463

[B29] Henning R. J. (2021). Cardiovascular exosomes and MicroRNAs in cardiovascular physiology and pathophysiology. J. Cardiovasc. Transl. Res. 14, 195–212. 10.1007/s12265-020-10040-5 32588374

[B30] Hernández J. S. Barreto-Torres G. Kuznetsov A. V. Khuchua Z. Javadov S. (2014). Crosstalk between AMPK activation and angiotensin II-induced hypertrophy in cardiomyocytes: The role of mitochondria. J. Cell Mol. Med. 18, 709–720. 10.1111/jcmm.12220 24444314 PMC3981893

[B31] Horman S. Beauloye C. Vanoverschelde J. L. Bertrand L. (2012). AMP-activated protein kinase in the control of cardiac metabolism and remodeling. Curr. heart Fail. Rep. 9, 164–173. 10.1007/s11897-012-0102-z 22767403

[B32] Hu C. Zhang X. Song P. Yuan Y. P. Kong C. Y. Wu H. M. (2020). Meteorin-like protein attenuates doxorubicin-induced cardiotoxicity via activating cAMP/PKA/SIRT1 pathway. Redox Biol. 37, 101747. 10.1016/j.redox.2020.101747 33045622 PMC7558217

[B33] Huang Y. Kwan K. K. L. Leung K. W. Yao P. Wang H. Dong T. T. (2019). Ginseng extracts modulate mitochondrial bioenergetics of live cardiomyoblasts: A functional comparison of different extraction solvents. J. ginseng Res. 43, 517–526. 10.1016/j.jgr.2018.02.002 31695560 PMC6823796

[B34] Jia W. Bai T. Zeng J. Niu Z. Fan D. Xu X. (2021). Combined administration of metformin and atorvastatin attenuates diabetic cardiomyopathy by inhibiting inflammation, apoptosis, and oxidative stress in type 2 diabetic mice. Front. cell Dev. Biol. 9, 634900. 10.3389/fcell.2021.634900 33718370 PMC7945946

[B35] Jiang Q. Lu M. Li J. Zhu Z. (2021). Ginkgolide B protects cardiomyocytes from angiotensin II-induced hypertrophy via regulation of autophagy through SIRT1-FoxO1. Cardiovasc. Ther. 2021, 5554569. 10.1155/2021/5554569 34257705 PMC8245256

[B36] Karbasforooshan H. Karimi G. (2017). The role of SIRT1 in diabetic cardiomyopathy. Biomed. Pharmacother. = Biomedecine Pharmacother. 90, 386–392. 10.1016/j.biopha.2017.03.056 28380414

[B37] Kerr J. F. Wyllie A. H. Currie A. R. (1972). Apoptosis: A basic biological phenomenon with wide-ranging implications in tissue kinetics. Br. J. cancer 26, 239–257. 10.1038/bjc.1972.33 4561027 PMC2008650

[B38] Kim D. H. Park C. H. Park D. Choi Y. J. Park M. H. Chung K. W. (2014). Ginsenoside Rc modulates Akt/FoxO1 pathways and suppresses oxidative stress. Archives pharmacal Res. 37, 813–820. 10.1007/s12272-013-0223-2 23918648

[B39] Kim M. J. An H. J. Kim D. H. Lee B. Lee H. J. Ullah S. (2018). Novel SIRT1 activator MHY2233 improves glucose tolerance and reduces hepatic lipid accumulation in db/db mice. Bioorg. Med. Chem. Lett. 28, 684–688. 10.1016/j.bmcl.2018.01.021 29402742

[B40] Kim S. C. Kim Y. H. Son S. W. Moon E. Y. Pyo S. Um S. H. (2015). Fisetin induces Sirt1 expression while inhibiting early adipogenesis in 3T3-L1 cells. Biochem. biophysical Res. Commun. 467, 638–644. 10.1016/j.bbrc.2015.10.094 26499075

[B41] Kimura Y. Izumiya Y. Araki S. Yamamura S. Hanatani S. Onoue Y. (2021). Sirt7 deficiency attenuates neointimal formation following vascular injury by modulating vascular Smooth muscle cell proliferation. Circulation J. official J. Jpn. Circulation Soc. 85, 2232–2240. 10.1253/circj.CJ-20-0936 33678753

[B42] Laddha A. P. Kulkarni Y. A. (2021). Daidzein mitigates myocardial injury in streptozotocin-induced diabetes in rats. Life Sci. 284, 119664. 10.1016/j.lfs.2021.119664 34090859

[B43] Li S. Huang Q. Zhang L. Qiao X. Zhang Y. Tang F. (2019a). Effect of CAPE-pNO(2) against type 2 diabetes mellitus via the AMPK/GLUT4/GSK3β/PPARα pathway in HFD/STZ-induced diabetic mice. Eur. J. Pharmacol. 853, 1–10. 10.1016/j.ejphar.2019.03.027 30885574

[B44] Li S. Zhu Z. Xue M. Yi X. Liang J. Niu C. (2019b). Fibroblast growth factor 21 protects the heart from angiotensin II-induced cardiac hypertrophy and dysfunction via SIRT1. Biochimica biophysica acta. Mol. basis Dis. 1865, 1241–1252. 10.1016/j.bbadis.2019.01.019 30677512

[B45] Li Y. Meng X. Wang W. Liu F. Hao Z. Yang Y. (2017). Cardioprotective effects of SIRT6 in a mouse model of transverse aortic constriction-induced heart failure. Front. physiology 8, 394. 10.3389/fphys.2017.00394 PMC5468374 28659816

[B46] Lin X. Liu C. Wang T. Wang H. Shao Z. (2019). Sirt1 in the regulation of interferon gamma in severe aplastic anemia. Acta Haematol. 142, 142–148. 10.1159/000497404 31141802

[B47] Liu J. Ai Y. Niu X. Shang F. Li Z. Liu H. (2020). Taurine protects against cardiac dysfunction induced by pressure overload through SIRT1-p53 activation. Chemico-biological Interact. 317, 108972. 10.1016/j.cbi.2020.108972 32017914

[B48] Liu W. Chen P. Deng J. Lv J. Liu J. (2017). Resveratrol and polydatin as modulators of Ca(2+) mobilization in the cardiovascular system. Ann. N. Y. Acad. Sci. 1403, 82–91. 10.1111/nyas.13386 28665033

[B49] Liu Z. H. Zhang Y. Wang X. Fan X. F. Zhang Y. Li X. (2019). SIRT1 activation attenuates cardiac fibrosis by endothelial-to-mesenchymal transition. Biomed. Pharmacother. 118, 109227. 10.1016/j.biopha.2019.109227 31351433

[B50] Lohanathan B. P. Rathinasamy B. Huang C. Y. Viswanadha V. P. (2022). Neferine attenuates doxorubicin-induced fibrosis and hypertrophy in H9c2 cells. J. Biochem. Mol. Toxicol. 36, e23054. 10.1002/jbt.23054 35347819

[B51] Lu B. Zhang D. Wang X. Lin D. Chen Y. Xu X. (2021a). Targeting SIRT1 to inhibit the proliferation of multiple myeloma cells. Oncol. Lett. 21, 306. 10.3892/ol.2021.12567 33732382 PMC7905587

[B52] Lu H. Jia C. Wu D. Jin H. Lin Z. Pan J. (2021b). Fibroblast growth factor 21 (FGF21) alleviates senescence, apoptosis, and extracellular matrix degradation in osteoarthritis via the SIRT1-mTOR signaling pathway. Cell death Dis. 12, 865. 10.1038/s41419-021-04157-x 34556628 PMC8460788

[B53] Luo G. Jian Z. Zhu Y. Zhu Y. Chen B. Ma R. (2019). Sirt1 promotes autophagy and inhibits apoptosis to protect cardiomyocytes from hypoxic stress. Int. J. Mol. Med. 43, 2033–2043. 10.3892/ijmm.2019.4125 30864731 PMC6443335

[B54] Luo Y. X. Tang X. An X. Z. Xie X. M. Chen X. F. Zhao X. (2017). SIRT4 accelerates Ang II-induced pathological cardiac hypertrophy by inhibiting manganese superoxide dismutase activity. Eur. heart J. 38, 1389–1398. 10.1093/eurheartj/ehw138 27099261

[B55] Ma W. Zhao X. Wang K. Liu J. Huang G. (2018). Dichloroacetic acid (DCA) synergizes with the SIRT2 inhibitor Sirtinol and AGK2 to enhance anti-tumor efficacy in non-small cell lung cancer. Cancer Biol. Ther. 19, 835–846. 10.1080/15384047.2018.1480281 30067423 PMC6154844

[B56] Mao S. Chen P. Li T. Guo L. Zhang M. (2018). Tongguan capsule mitigates post-myocardial infarction remodeling by promoting autophagy and inhibiting apoptosis: Role of Sirt1. Front. physiology 9, 589. 10.3389/fphys.2018.00589 PMC5972280 29872406

[B57] Milne J. C. Lambert P. D. Schenk S. Carney D. P. Smith J. J. Gagne D. J. (2007). Small molecule activators of SIRT1 as therapeutics for the treatment of type 2 diabetes. Nature 450, 712–716. 10.1038/nature06261 18046409 PMC2753457

[B58] Minor R. K. Baur J. A. Gomes A. P. Ward T. M. Csiszar A. Mercken E. M. (2011). SRT1720 improves survival and healthspan of obese mice. Sci. Rep. 1, 70. 10.1038/srep00070 22355589 PMC3216557

[B59] Modi S. Yaluri N. Kokkola T. Laakso M. (2017). Plant-derived compounds strigolactone GR24 and pinosylvin activate SIRT1 and enhance glucose uptake in rat skeletal muscle cells. Sci. Rep. 7, 17606. 10.1038/s41598-017-17840-x 29242624 PMC5730588

[B60] Oka S. Alcendor R. Zhai P. Park J. Y. Shao D. Cho J. (2011). PPARα-Sirt1 complex mediates cardiac hypertrophy and failure through suppression of the ERR transcriptional pathway. Cell metab. 14, 598–611. 10.1016/j.cmet.2011.10.001 22055503 PMC3217210

[B61] Osataphan S. Macchi C. Singhal G. Chimene-Weiss J. Sales V. Kozuka C. (2019). SGLT2 inhibition reprograms systemic metabolism via FGF21-dependent and -independent mechanisms. JCI insight 4, e123130. 10.1172/jci.insight.123130 30843877 PMC6483601

[B62] Ota H. Tokunaga E. Chang K. Hikasa M. Iijima K. Eto M. (2006). Sirt1 inhibitor, Sirtinol, induces senescence-like growth arrest with attenuated Ras-MAPK signaling in human cancer cells. Oncogene 25, 176–185. 10.1038/sj.onc.1209049 16170353

[B63] Pagans S. Pedal A. North B. J. Kaehlcke K. Marshall B. L. Dorr A. (2005). SIRT1 regulates HIV transcription via Tat deacetylation. PLoS Biol. 3, e41. 10.1371/journal.pbio.0030041 15719057 PMC546329

[B64] Palojoki E. Saraste A. Eriksson A. Pulkki K. Kallajoki M. Voipio-Pulkki L. M. (2001). Cardiomyocyte apoptosis and ventricular remodeling after myocardial infarction in rats. Am. J. physiology. Heart circulatory physiology 280, H2726–H2731. 10.1152/ajpheart.2001.280.6.H2726 11356629

[B65] Planavila A. Dominguez E. Navarro M. Vinciguerra M. Iglesias R. Giralt M. (2012). Dilated cardiomyopathy and mitochondrial dysfunction in sirt1-deficient mice: A role for sirt1-mef2 in adult heart. J. Mol. Cell. Cardiol. 53, 521–531. 10.1016/j.yjmcc.2012.07.019 22986367

[B66] Planavila A. Iglesias R. Giralt M. Villarroya F. (2011). Sirt1 acts in association with PPARα to protect the heart from hypertrophy, metabolic dysregulation, and inflammation. Cardiovasc. Res. 90, 276–284. 10.1093/cvr/cvq376 21115502

[B67] Price N. L. Gomes A. P. Ling A. J. Duarte F. V. Martin-Montalvo A. North B. J. (2012). SIRT1 is required for AMPK activation and the beneficial effects of resveratrol on mitochondrial function. Cell metab. 15, 675–690. 10.1016/j.cmet.2012.04.003 22560220 PMC3545644

[B68] Qiu L. Luo Y. Chen X. (2018). Quercetin attenuates mitochondrial dysfunction and biogenesis via upregulated AMPK/SIRT1 signaling pathway in OA rats. Biomed. Pharmacother. 103, 1585–1591. 10.1016/j.biopha.2018.05.003 29864946

[B69] Rababa'h A. M. Guillory A. N. Mustafa R. Hijjawi T. (2018). Oxidative stress and cardiac remodeling: An updated edge. Curr. Cardiol. Rev. 14, 53–59. 10.2174/1573403x14666180111145207 29332590 PMC5872263

[B70] Ren B. C. Zhang Y. F. Liu S. S. Cheng X. J. Yang X. Cui X. G. (2020). Curcumin alleviates oxidative stress and inhibits apoptosis in diabetic cardiomyopathy via Sirt1-Foxo1 and PI3K-Akt signalling pathways. J. Cell Mol. Med. 24, 12355–12367. 10.1111/jcmm.15725 32961025 PMC7687015

[B71] Ren B. Feng J. Yang N. Guo Y. Chen C. Qin Q. (2021). Ginsenoside Rg3 attenuates angiotensin II-induced myocardial hypertrophy through repressing NLRP3 inflammasome and oxidative stress via modulating SIRT1/NF-κB pathway. Int. Immunopharmacol. 98, 107841. 10.1016/j.intimp.2021.107841 34153662

[B72] Ren J. Yang L. Zhu L. Xu X. Ceylan A. F. Guo W. (2017). Akt2 ablation prolongs life span and improves myocardial contractile function with adaptive cardiac remodeling: Role of sirt1-mediated autophagy regulation. Aging cell 16, 976–987. 10.1111/acel.12616 28681509 PMC5595687

[B73] Rodgers J. T. Lerin C. Haas W. Gygi S. P. Spiegelman B. M. Puigserver P. (2005). Nutrient control of glucose homeostasis through a complex of PGC-1alpha and SIRT1. Nature 434, 113–118. 10.1038/nature03354 15744310

[B74] Safari F. Shekarforoosh S. Hashemi T. Namvar Aghdash S. Fekri A. Safari F. (2017). Sirtinol abrogates late phase of cardiac ischemia preconditioning in rats. J. physiological Sci. 67, 515–522. 10.1007/s12576-016-0483-y PMC10717902 27677982

[B75] Sciarretta S. Maejima Y. Zablocki D. Sadoshima J. (2018). The role of autophagy in the heart. Annu. Rev. physiology 80, 1–26. 10.1146/annurev-physiol-021317-121427 29068766

[B76] Shah A. K. Bhullar S. K. Elimban V. Dhalla N. S. (2021). Oxidative stress as A mechanism for functional alterations in cardiac hypertrophy and heart failure. Antioxidants (Basel) 10, 931. 10.3390/antiox10060931 34201261 PMC8228897

[B77] Shirakabe A. Zhai P. Ikeda Y. Saito T. Maejima Y. Hsu C. P. (2016). Drp1-Dependent mitochondrial autophagy plays a protective role against pressure overload-induced mitochondrial dysfunction and heart failure. Circulation 133, 1249–1263. 10.1161/circulationaha.115.020502 26915633 PMC4811679

[B78] Snopek L. Mlcek J. Sochorova L. Baron M. Hlavacova I. Jurikova T. (2018). Contribution of Red Wine Consumption to Human Health Protection. Mol 23, 1684. 10.3390/molecules23071684 PMC6099584 29997312

[B79] Sun M. Zhai M. Zhang N. Wang R. Liang H. Han Q. (2018). MicroRNA-148b-3p is involved in regulating hypoxia/reoxygenation-induced injury of cardiomyocytes *in vitro* through modulating SIRT7/p53 signaling. Chemico-biological Interact. 296, 211–219. 10.1016/j.cbi.2018.10.003 30308185

[B80] Sun X. Duan J. Gong C. Feng Y. Hu J. Gu R. (2022a). Colchicine ameliorates dilated cardiomyopathy via SIRT2-mediated suppression of NLRP3 inflammasome activation. J. Am. Heart Assoc. 11, e025266. 10.1161/jaha.122.025266 35766262 PMC9333380

[B81] Sun Y. Yang Y. M. Hu Y. Y. Ouyang L. Sun Z. H. Yin X. F. (2022b). Inhibition of nuclear deacetylase Sirtuin-1 induces mitochondrial acetylation and calcium overload leading to cell death. Redox Biol. 53, 102334. 10.1016/j.redox.2022.102334 35636016 PMC9142701

[B82] Tang X. Chen X. F. Wang N. Y. Wang X. M. Liang S. T. Zheng W. (2017). SIRT2 acts as a cardioprotective deacetylase in pathological cardiac hypertrophy. Circulation 136, 2051–2067. 10.1161/circulationaha.117.028728 28947430 PMC5698109

[B83] Tham Y. K. Bernardo B. C. Ooi J. Y. Weeks K. L. Mcmullen J. R. (2015). Pathophysiology of cardiac hypertrophy and heart failure: Signaling pathways and novel therapeutic targets. Archives Toxicol. 89, 1401–1438. 10.1007/s00204-015-1477-x 25708889

[B84] Trapp J. Meier R. Hongwiset D. Kassack M. U. Sippl W. Jung M. (2007). Structure-activity studies on suramin analogues as inhibitors of NAD+-dependent histone deacetylases (sirtuins). ChemMedChem 2, 1419–1431. 10.1002/cmdc.200700003 17628866

[B85] Ueno T. Endo S. Saito R. Hirose M. Hirai S. Suzuki H. (2013). The sirtuin inhibitor tenovin-6 upregulates death receptor 5 and enhances cytotoxic effects of 5-fluorouracil and oxaliplatin in colon cancer cells. Oncol. Res. 21, 155–164. 10.3727/096504013x13854886566598 24512730

[B86] Um J. H. Park S. J. Kang H. Yang S. Foretz M. Mcburney M. W. (2010). AMP-activated protein kinase-deficient mice are resistant to the metabolic effects of resveratrol. Diabetes 59, 554–563. 10.2337/db09-0482 19934007 PMC2828647

[B87] Vahtola E. Louhelainen M. Merasto S. Martonen E. Penttinen S. Aahos I. (2008). Forkhead class O transcription factor 3a activation and Sirtuin1 overexpression in the hypertrophied myocardium of the diabetic Goto-Kakizaki rat. J. Hypertens. 26, 334–344. 10.1097/HJH.0b013e3282f293c8 18192848

[B88] Vakhrusheva O. Smolka C. Gajawada P. Kostin S. Boettger T. Kubin T. (2008). Sirt7 increases stress resistance of cardiomyocytes and prevents apoptosis and inflammatory cardiomyopathy in mice. Circulation Res. 102, 703–710. 10.1161/circresaha.107.164558 18239138

[B89] Veeroju S. Mamazhakypov A. Rai N. Kojonazarov B. Nadeau V. Breuils-Bonnet S. (2020). Effect of p53 activation on experimental right ventricular hypertrophy. PloS one 15, e0234872. 10.1371/journal.pone.0234872 32559203 PMC7304610

[B90] Venkatasubramanian S. Noh R. M. Daga S. Langrish J. P. Joshi N. V. Mills N. L. (2013). Cardiovascular effects of a novel SIRT1 activator, SRT2104, in otherwise healthy cigarette smokers. J. Am. Heart Assoc. 2, e000042. 10.1161/jaha.113.000042 23770971 PMC3698759

[B91] Waldman M. Cohen K. Yadin D. Nudelman V. Gorfil D. Laniado-Schwartzman M. (2018). Regulation of diabetic cardiomyopathy by caloric restriction is mediated by intracellular signaling pathways involving 'SIRT1 and PGC-1α. Cardiovasc. Diabetol. 17, 111. 10.1186/s12933-018-0754-4 30071860 PMC6090985

[B92] Wang T. Li X. Sun S. L. (2020a). EX527, a Sirt-1 inhibitor, induces apoptosis in glioma via activating the p53 signaling pathway. Anti-cancer drugs 31, 19–26. 10.1097/cad.0000000000000824 31490284

[B93] Wang Y. Liang X. Chen Y. Zhao X. (2016). Screening SIRT1 activators from medicinal plants as bioactive compounds against oxidative damage in mitochondrial function. Oxidative Med. Cell. Longev. 2016, 4206392. 10.1155/2016/4206392 PMC4766345 26981165

[B94] Wang Y. Xie Z. Jiang N. Wu Z. Xue R. Dong B. (2020b). Hispidulin attenuates cardiac hypertrophy by improving mitochondrial dysfunction. Front. Cardiovasc. Med. 7, 582890. 10.3389/fcvm.2020.582890 33324687 PMC7726192

[B95] Wu S. Lan J. Li L. Wang X. Tong M. Fu L. (2021). Sirt6 protects cardiomyocytes against doxorubicin-induced cardiotoxicity by inhibiting P53/Fas-dependent cell death and augmenting endogenous antioxidant defense mechanisms. Cell Biol. Toxicol. 2021. 10.1007/s10565-021-09649-2 34713381

[B96] Wu X. He L. Cai Y. Zhang G. He Y. Zhang Z. (2013). Induction of autophagy contributes to the myocardial protection of valsartan against ischemia-reperfusion injury. Mol. Med. Rep. 8, 1824–1830. 10.3892/mmr.2013.1708 24084854

[B97] Xie Z. Lau K. Eby B. Lozano P. He C. Pennington B. (2011). Improvement of cardiac functions by chronic metformin treatment is associated with enhanced cardiac autophagy in diabetic OVE26 mice. Diabetes 60, 1770–1778. 10.2337/db10-0351 21562078 PMC3114402

[B98] Xin T. Lu C. (2020). SirT3 activates AMPK-related mitochondrial biogenesis and ameliorates sepsis-induced myocardial injury. Aging 12, 16224–16237. 10.18632/aging.103644 32721927 PMC7485737

[B99] Xing Y. Q. Li A. Yang Y. Li X. X. Zhang L. N. Guo H. C. (2018). The regulation of FOXO1 and its role in disease progression. Life Sci. 193, 124–131. 10.1016/j.lfs.2017.11.030 29158051

[B100] Yamamura S. Izumiya Y. Araki S. Nakamura T. Kimura Y. Hanatani S. (2020). Cardiomyocyte sirt (sirtuin) 7 ameliorates stress-induced cardiac hypertrophy by interacting with and deacetylating GATA4. Hypertens. 75, 98–108. 10.1161/hypertensionaha.119.13357 31735083

[B101] Yang Q. Y. Lai X. D. Ouyang J. Yang J. D. (2018). Effects of Ginsenoside Rg3 on fatigue resistance and SIRT1 in aged rats. Toxicology 409, 144–151. 10.1016/j.tox.2018.08.010 30144466

[B102] Yang X. Jin Z. Lin D. Shen T. Zhang J. Li D. (2022). FGF21 alleviates acute liver injury by inducing the SIRT1-autophagy signalling pathway. J. Cell. Mol. Med. 26, 868–879. 10.1111/jcmm.17144 34984826 PMC8817117

[B103] Yar Saglam A. S. Yilmaz A. Onen H. I. Alp E. Kayhan H. Ekmekci A. (2016). HDAC inhibitors, MS-275 and salermide, potentiates the anticancer effect of EF24 in human pancreatic cancer cells. EXCLI J. 15, 246–255. 10.17179/excli2016-186 27330528 PMC4908665

[B104] Ying Y. Jiang C. Zhang M. Jin J. Ge S. Wang X. (2019). Phloretin protects against cardiac damage and remodeling via restoring SIRT1 and anti-inflammatory effects in the streptozotocin-induced diabetic mouse model. Aging 11, 2822–2835. 10.18632/aging.101954 31076562 PMC6535073

[B105] Yu L. M. Dong X. Xue X. D. Xu S. Zhang X. Xu Y. L. (2021). Melatonin attenuates diabetic cardiomyopathy and reduces myocardial vulnerability to ischemia-reperfusion injury by improving mitochondrial quality control: Role of SIRT6. J. pineal Res. 70, e12698. 10.1111/jpi.12698 33016468

[B106] Yu L. She T. Li M. Shi C. Han L. Cheng M. (2013). Tetramethylpyrazine inhibits angiotensin II-induced cardiomyocyte hypertrophy and tumor necrosis factor-α secretion through an NF-κB-dependent mechanism. Int. J. Mol. Med. 32, 717–722. 10.3892/ijmm.2013.1436 23842595

[B107] Yuan H. Wang Z. Li L. Zhang H. Modi H. Horne D. (2012). Activation of stress response gene SIRT1 by BCR-ABL promotes leukemogenesis. Blood 119, 1904–1914. 10.1182/blood-2011-06-361691 22207735 PMC3293644

[B108] Yuan Y. P. Ma Z. G. Zhang X. Xu S. C. Zeng X. F. Yang Z. (2018). CTRP3 protected against doxorubicin-induced cardiac dysfunction, inflammation and cell death via activation of Sirt1. J. Mol. Cell. Cardiol. 114, 38–47. 10.1016/j.yjmcc.2017.10.008 29061338

[B109] Zeng G. Liu H. Wang H. (2018). Amelioration of myocardial ischemia-reperfusion injury by SIRT4 involves mitochondrial protection and reduced apoptosis. Biochem. biophysical Res. Commun. 502, 15–21. 10.1016/j.bbrc.2018.05.113 29777709

[B110] Zhan H. Huang F. Niu Q. Jiao M. Han X. Zhang K. (2021). Downregulation of miR-128 ameliorates Ang II-induced cardiac remodeling via SIRT1/PIK3R1 multiple targets. Oxidative Med. Cell. Longev. 2021, 8889195. 10.1155/2021/8889195 PMC8505057 34646427

[B111] Zhang B. Zhai M. Li B. Liu Z. Li K. Jiang L. (2018). Honokiol ameliorates myocardial ischemia/reperfusion injury in type 1 diabetic rats by reducing oxidative stress and apoptosis through activating the SIRT1-nrf2 signaling pathway. Oxidative Med. Cell. Longev. 2018, 3159801. 10.1155/2018/3159801 PMC5838504 29675132

[B112] Zhang C. Li C. Chen S. Li Z. Ma L. Jia X. (2017a). Hormetic effect of panaxatriol saponins confers neuroprotection in PC12 cells and zebrafish through PI3K/AKT/mTOR and AMPK/SIRT1/FOXO3 pathways. Sci. Rep. 7, 41082. 10.1038/srep41082 28112228 PMC5253660

[B113] Zhang J. Cheng Y. Gu J. Wang S. Zhou S. Wang Y. (2016). Fenofibrate increases cardiac autophagy via FGF21/SIRT1 and prevents fibrosis and inflammation in the hearts of Type 1 diabetic mice. Clin. Sci. (Lond) 130, 625–641. 10.1042/cs20150623 26795437

[B114] Zhang J. Huang L. Shi X. Yang L. Hua F. Ma J. (2020). Metformin protects against myocardial ischemia-reperfusion injury and cell pyroptosis via AMPK/NLRP3 inflammasome pathway. Aging 12, 24270–24287. 10.18632/aging.202143 33232283 PMC7762510

[B115] Zhang M. Sui W. Xing Y. Cheng J. Cheng C. Xue F. (2021a). Angiotensin IV attenuates diabetic cardiomyopathy via suppressing FoxO1-induced excessive autophagy, apoptosis and fibrosis. Theranostics 11, 8624–8639. 10.7150/thno.48561 34522203 PMC8419053

[B116] Zhang N. Hu Y. Ding C. Zeng W. Shan W. Fan H. (2017b). Salvianolic acid B protects against chronic alcoholic liver injury via SIRT1-mediated inhibition of CRP and ChREBP in rats. Toxicol. Lett. 267, 1–10. 10.1016/j.toxlet.2016.12.010 27989594

[B117] Zhang S. Wu P. Liu J. Du Y. Yang Z. (2021b). Roflumilast attenuates doxorubicin-induced cardiotoxicity by targeting inflammation and cellular senescence in cardiomyocytes mediated by SIRT1. Drug Des. Dev. Ther. 15, 87–97. 10.2147/dddt.S269029 PMC7810683 33469262

[B118] Zhang Y. Mi S. L. Hu N. Doser T. A. Sun A. Ge J. (2014). Mitochondrial aldehyde dehydrogenase 2 accentuates aging-induced cardiac remodeling and contractile dysfunction: Role of AMPK, Sirt1, and mitochondrial function. Free Radic. Biol. Med. 71, 208–220. 10.1016/j.freeradbiomed.2014.03.018 24675227 PMC4068748

[B119] Zhao L. Qi Y. Xu L. Tao X. Han X. Yin L. (2018a). MicroRNA-140-5p aggravates doxorubicin-induced cardiotoxicity by promoting myocardial oxidative stress via targeting Nrf2 and Sirt2. Redox Biol. 15, 284–296. 10.1016/j.redox.2017.12.013 29304479 PMC5975069

[B120] Zhao L. Tao X. Qi Y. Xu L. Yin L. Peng J. (2018b). Protective effect of dioscin against doxorubicin-induced cardiotoxicity via adjusting microRNA-140-5p-mediated myocardial oxidative stress. Redox Biol. 16, 189–198. 10.1016/j.redox.2018.02.026 29524841 PMC5953242

[B121] Zheng C. B. Gao W. C. Xie M. Li Z. Ma X. Song W. (2021). Ang II promotes cardiac autophagy and hypertrophy via orai1/STIM1. Front. Pharmacol. 12, 622774. 10.3389/fphar.2021.622774 34079454 PMC8165566

